# Multiscale Analysis of Defect Structures in Single-Crystalline CMSX-4 Superalloys

**DOI:** 10.3390/ma18081819

**Published:** 2025-04-16

**Authors:** Robert Paszkowski, Sławomir Kołodziej, Mirosława Pawlyta, Beata Chrząszcz

**Affiliations:** 1 Institute of Materials Engineering, University of Silesia in Katowice, 1A 75 Pułku Piechoty St., 41-500 Chorzów, Poland; beata.chrzaszcz@us.edu.pl; 2 Materials Research Laboratory, Faculty of Mechanical Engineering, Silesian University of Technology, Konarskiego 18 A, 44-100 Gliwice, Poland; miroslawa.pawlyta@polsl.pl; 3 Electron Microscopy Facility, School of Chemistry, University of St Andrews, St Andrews KY16 9AJ, UK

**Keywords:** nickel-based superalloy, Bridgman technique, X-ray topography, positron annihilation lifetime spectroscopy (PALS), transmission electron microscopy (TEM), dendrite array, defects, vacancy-type defects, low-angle boundaries

## Abstract

An analysis of defects creation in the vicinity of the selector-root connection plane in single-crystalline turbine blades made of CMSX-4 Ni-base superalloy was performed using several experimental methods. A coupling of scanning electron microscopy and X-ray diffraction topography allowed the visualization of dendritic arrays and surface defects in the root part of the blades. As a result, contrast inversions and areas where internal stresses occur were observed. The defects on a microscopic scale were characterized using positron annihilation lifetime spectroscopy and transmission electron microscopy. The registered positron lifetimes, above 0.5 ns, beyond the range characteristic for defects generally reported in metals and their alloys suggest the presence extremely large void type defects. Herein, we have identified large defects, ca. 2–5 nm in diameter, formed due to the contraction of fluid metal, captured in inter-dendritic regions during the liquid-to-solid transition. This work is a precursor to the almost untouched area of the discussion of lifetimes characteristic for positron bound states, called positronium (>0.5 ns) in relation to the morphology of void-type defects in single-crystalline superalloys.

## 1. Introduction

**Figure A1:**
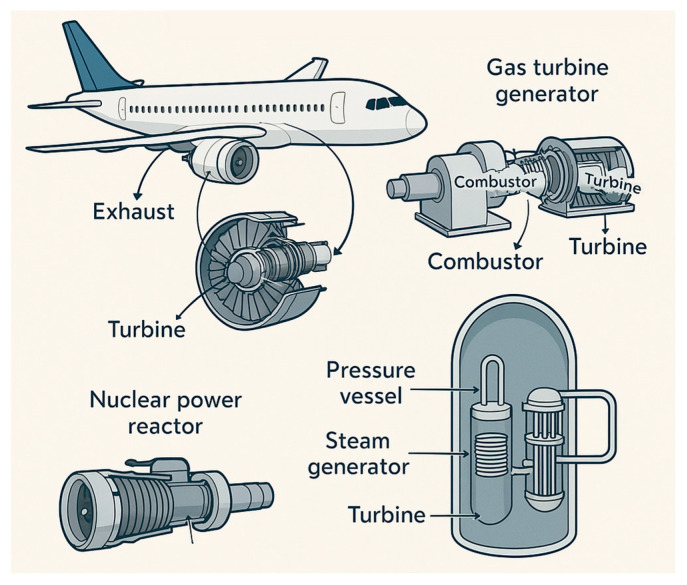
Diagram illustrating the components made of superalloys used in aerospace devices and energy sector components. The labelled parts in a commercial aircraft turbine engine and energy sector components like a gas turbine generator and nuclear power reactor can be observed.

Nickel-based superalloys are the most commonly used materials for manufacturing single-crystal turbine components and are widely utilized in the aerospace and energy sectors—Figure A1 in Appendix A. These materials fulfil numerous requirements, including resistance to high temperatures and corrosion, as well as high mechanical strength under complex loads [1,2,3,4]. One of the most widely used nickel-based superalloys is CMSX-4, which belongs to the 2nd generation of single-crystalline superalloys. The alloy consists of ten primary alloying elements—chemical composition is given in Table A1 in Appendix A. Mechanical and physical properties of CMSX-4 are shown in Table A2 in Appendix A. The structure consists of two phases: γ-phase in the form of a thin channels net as the matrix, and γ′ cubic crystals about 1 µm or less in size as reinforcement [1,2,3,4].

Single-crystal turbine blades are typically manufactured using ceramic moulds through directional crystallization via the Bridgman method. This approach enables the production of casts with high homogeneity, featuring a parallel arrangement of dendrites that share the same [001] crystal orientation. The orientation is determined by a narrow diameter selector connected to a wide root and cut off at the next production stage. During crystallization, transitions from a narrow selector to a wide root can lead to the formation of various defects, such as low-angle boundaries (LABs) [5,6], vacancies [7], and casting stresses [8,9]. However, Krawczyk et al. [10] investigated that the defects inherited during crystallization from the root to the blade’s airfoil persist even after heat treatment. The concentration of vacancy-type defects in multicomponent technical superalloys containing numerous alloying elements is particularly significant, as it is related to the kinetics of diffusion processes [11,12]. Furthermore, Schenk et al. [13] highlighted that the density of dislocations and vacancy-type defects plays a critical role in the creep processes of nickel-based single-crystal superalloys. As a result, it is imperative to investigate the distribution of defects in the root layers adjacent to the selector-root connection and to examine their formation mechanisms.

The X-ray diffraction topography method facilitates the analysis of defects occurring during individual dendrites’ lateral growth and the dendrite array’s formation. By utilizing a divergent beam emitted from a microfocus X-ray tube, this technique can cover the entire surface of the sample, which oscillates during exposure. This method enables the visualization of small misorientations between neighboring regions and adjacent dendrites (measured in angular minutes) that cannot be detected by other methods, such as electron backscatter diffraction (EBSD), over large areas (ranging from several to dozens of cm²) of the blade. This method is also utilized to examine the variations in the types and densities of defects, including dislocations, vacancies, and low-angle boundaries, across the macroscopic areas of the single-crystal turbine blade [6,9,14]. Nonetheless, all these changes at the microscopic level can be analyzed using positron annihilation lifetime spectroscopy (PALS). This technique is a powerful and promising nondestructive method for quality control of technologically significant materials across various scientific and technological fields. PALS measures the time elapsed between the implantation of a positron into the material and the emission of annihilation radiation. When a positron becomes trapped in a defect, its lifetime increases in relation to the size of the defect due to the locally decreased electron density, which results in a longer positron lifetime. Therefore, the sensitive method, as the PALS technique is, allows to determine the size and concentration of defects like vacancies, vacancy clusters, etc. [15,16]

**Figure 1:**
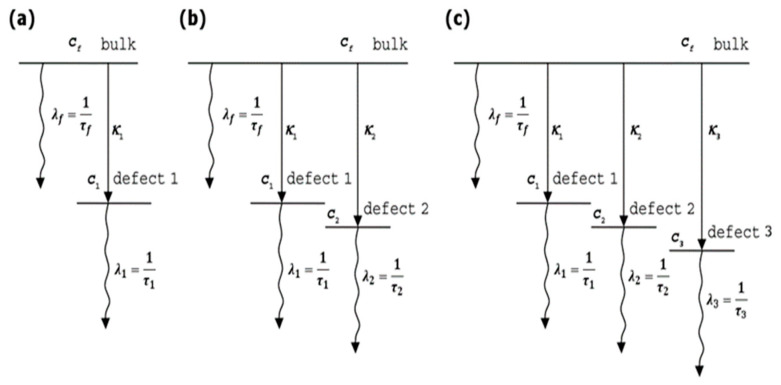
The scheme of the positron annihilation processes of the (**a**) two-, (**b**) three- and (**c**) four-state trapping model. c_f_, c_1_, c_2_, and c_3_ describe the probability of finding the positron in bulk, the first, second, or third type of defects.

Well suited for positron annihilation lifetime analysis is the LT10 software written by Giebel and Kansy with an intuitive user interface [17,18]. An unquestionable advantage of this program is the possibility of fitting a couple of spectra in a series. The simultaneous fitting significantly reduces the number of freedom degrees in the numerical analysis of PAL spectra because some parameters can have common values for all spectra in series. LT10 provides many built-in theoretical models of positron annihilation, including the Simple Trapping Model (STM), which establishes the direct connection between the measured lifetime spectra and the defect’s type and concentration. The model assumes that a positron penetrating the sample may annihilate in bulk, or it can be trapped (without the possibility of de-trapping) and annihilated in a defined number of one, two or three different defect types. The scheme of the positron annihilation processes of all STM implemented in LT10 is presented in Figure 1.

Recent PALS studies of nickel-based compounds primarily focus on the characterization of defects in Ni_3_Al-based alloys and polycrystalline nickel-based superalloys [19,20,21,22,23]. Therefore, better characterization of these materials’ structures is of utmost importance.

For example in the work by Krawczyk et al. [10] on a CMSX-4 alloy, obtained with a 5 mm/min withdrawal rate, a positron lifetime τ = 1.5 ns registered for the as-cast material was related to micro-voids. However, the discussion of the origin of such long positron lifetimes in the material is lacking. Further in this work, the material was subjected to solution annealing in several technological steps at temperatures from 1277 °C to 1316 °C with subsequent aging (detailed description given in ref. [10]), which led to material’s homogenization and disappearance of this extremely long lifetime. Despite this, two shorter lifetimes were identified, close to 0.2 ns and 0.4 ns, which can be easily related to vacancy-associated structural defects. These lifetimes weren’t deconvoluted from the PALS spectra in the as-cast material.

Micro-porosity of as-cast nickel-based single-crystalline superalloys was also studied using optical microscopy by Anton and Giamei [24] and afterward by Gancarczyk et al. [25,26] and using scanning electron microscopy by Körner et al. [7]. The pores reported in these works are of micro-metric size (from 1 up to 200 µm^2^), and their origin is ascribed to the shrinkage of the liquid metal between the perpendicularly growing dendritic arms in the directional solidification process.

Therefore, the studies presented in this paper aim to explore the formation of defects in the root section of as-cast single-crystalline turbine blades produced from the commercial CMSX-4 nickel-based superalloy. The presented results for CMSX-4 are a continuation of the work started in 2019 about characterizing defects in other single-crystalline nickel-based superalloys—CMSX-6 [9].

## 2. Materials and Methods

**Figure 2:**
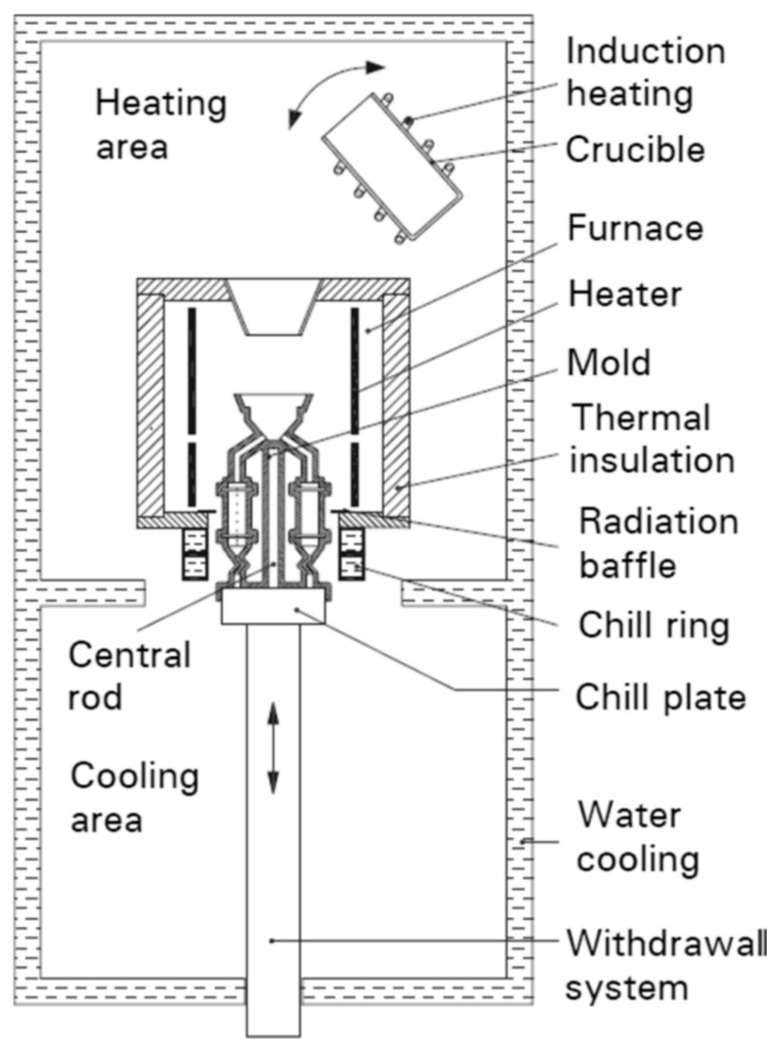
Scheme of the furnace for production of the nickel superalloy single-crystal blade taken from Ref. [28].

Single-crystal turbine blades made from CMSX-4 superalloy were fabricated at the Research and Development Laboratory for Aerospace Materials at Rzeszów University of Technology. The blades were produced using the directional Bridgman method with an axial orientation of the [001] type and a withdrawal rate of 1 mm/min in an industrial ALD furnace (ALD Vacuum Technologies Inc., East Windsor, CT, USA) [27]. The Bridgman method diagram is presented below (Figure 2) and was taken from Szeliga’s article [28].

**Figure 3:**
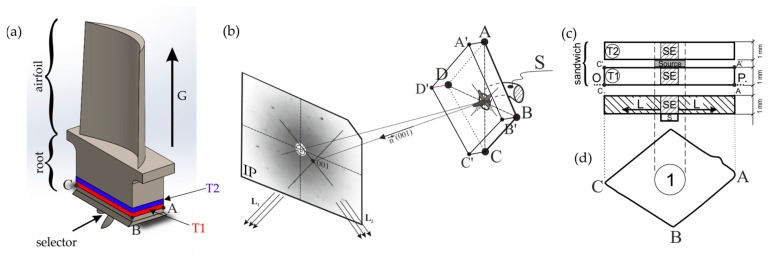
The location of the T1 and T2 samples within the root (**a**) and the arrangement of the Image Plate (IP) and sample during the formation of Laue patterns in reflection geometry (**b**). The specimens for PALS measurements are displayed in both perpendicular (**c**) and parallel (**d**) views relative to the turbine blade axis (G). The surfaces A′B′C′D′ and ABCD correspond to those from which the X-ray topograms were obtained. The positron source position for PALS measurements is indicated in area 1, while SE represents the selector extension area of the root, S denotes the spiral selector, and n indicates the vector of diffraction beams. L (L_1_ and L_2_) indicates the directions of lateral growth, and the O-P surface is designated for SEM observations. The hatched area was removed and excluded from the studies.

Specimens for the study were extracted from the roots of these turbine blades. To prepare the O-P surface for scanning electron microscopy (SEM) observations of the dendritic structure, mechanical polishing and chemical etching were employed. Two slices, designated T1 and T2, were cut perpendicular to the withdrawal direction (Z) near the selector-root connection plane (Figure 3a). Two plate-shaped specimens, T1 and T2, were prepared from the slices according to the selected crystallographic orientation characterized by the Laue method (Figure 3b). The T1 and T2 specimens were used to form a sandwich layout for PALS measurements (Figure 3c,d). X-ray topography was carried out from these samples to determine the PALS points.

### 2.1. X-Ray Diffraction Topography

The first step in the analysis of defect creation in the root part of as-cast single-crystalline turbine blades was the characterization of the specimens, carried out using scanning electron microscopy, Laue diffraction, and X-ray diffraction topography. The dendritic structure was visualized by scanning electron microscopy (SEM) JSM-6480 JEOL microscope (JEOL Ltd., Tokyo, Japan) with back-scattered electron imaging (BSE). The crystal orientation of the sample was determined by Laue diffraction in back-reflection geometry using the X-ray diffractometer of an XRT-100 system (EFG Freiberg Instruments, Freiberg, Germany).

**Figure 4:**
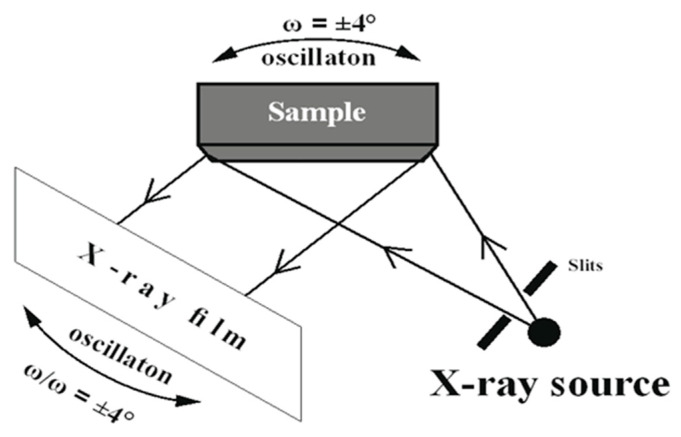
Scheme of X-ray diffraction topography.

The specimens, oriented by the Laue method, were analyzed by X-ray diffraction topography. X-ray topography was performed using a divergent beam from a CuKα X-ray source provided by the Panalytical Microfocus DY0601 diffractometer (Malvern PANalytical, Almelo, The Netherlands). The topograms were obtained using 113 type reflections during coupled sample and film oscillation around the Bragg angle (Figure 4). The procedures for obtaining and interpreting these topograms are detailed in Ref. [29].

### 2.2. Positron Annihilation Lifetime Spectroscopy

**Figure 5:**
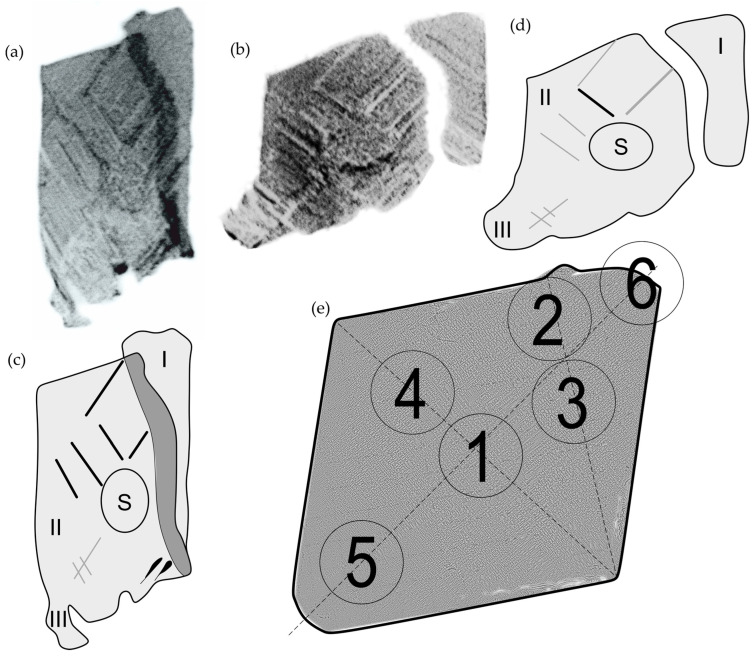
X-ray topograms obtained from the ABCD (**a**), A′B′C′D′ (**b**) surface using reflections 113 and the schemes of the topograms from Figure 5a (**c**) and Figure 5b (**d**) as well as a diagram of the distribution of points for PALS measurement (**e**). Below the diagram the distribution of the dendritic structure can be found, the description of which can be found earlier, and the drawing at a higher magnification is presented in Appendix A—Figure A2.

Positron lifetime measurements were performed under controlled conditions. Spectra were measured using a conventional “fast-fast” coincidence spectrometer equipped with plastic scintillators. The lifetime resolution approximated by the Gaussian function FWHM was found to be about 320 ps. The positron source (^22^NaCl) with the activity of 360 kBq embedded between two 7.6 µm Kapton foils was used. The source was placed between two pieces of the CMSX-4 alloy, at least 0.85 mm thick. Measurements were performed in a vacuum chamber, under a vacuum of 1 Pa, with a controlled temperature constant for each measurement of T = 293 K ± 0.2 K. The measurements were taken in 6 different areas (description below Figure 5).

Measurements in each area were performed at a 50 ns time scale. The particular spectra were taken every hour and composed by the special procedure accounting for the drift of the zero of the time scale. In this way, one characteristic spectrum containing at least 6 × 10^6^ total counts for each area was obtained. To give a view on the area of investigation at each point of measurement, we notice the ^22^NaCl droplet in the positron source was ca. 2 mm in diameter, and the total positron implantation range of positrons from the ^22^Na source into CMSX-4 alloys of density 8.7 g/cm^3^ [30] estimated based on the publication by Dryzek and Singleton [31] is ca. 200 µm. The measurements were repeated twice at each area irrespectively.

All these spectra for different areas of the given sample were analyzed together using LT10 software. The PALS spectra were fitted using the least-squares fitting procedure to the simple-state trapping model. The general equation of the fitting function *S* in this model is given by:

$$S=\left[I_{0}\lambda \exp\left(-\lambda t\right)+\sum_{i=1}^{N}I_{i}\lambda_{i}\exp\left(-\lambda_{i}t\right)\right]\cdot R$$

where: $\lambda_{f}=\tau_{f}^{-1}$, $\lambda_{i}=\tau_{i}^{-1}$, $\lambda =\lambda_{f}+\sum_{i=1}^{N}\kappa_{i}$, $I_{i}=\frac{\kappa_{i}}{\lambda -\lambda_{i}},I_{0}=1-\sum_{i=1}^{N}I_{i}$, while *N* equals the number of expected defect types—LT10 allows the use of *N* = 1 for the two-state trapping model, *N* = 2 for the three-state trapping model, and *N* = 3 for the four-state trapping model. The parameter *R* involves the instrumental resolution function and is a superposition of a view Gaussians.

In these equations *λ_f_* and *τ_f_* are the annihilation rate and lifetime in bulk material, *λ_i_* and *τ_i_* are the annihilation rate and lifetime in the *i*-th defect type, *κ_i_* is the trapping rate into the *i*-th defect, which are proportional to the defect concentrations, and *I*_0_ and *I_i_* are the intensities of the bulk and *i*-th-defect type component.

The concentration of the defects can be further calculated by dividing the positron trapping rates by the specific trapping rate of the analyzed defect type. The value of the second factor depends on the volume of the analyzed defect. Krsjak et al. [32], in the analysis of the positron trapping coefficient at nano-scale helium bubbles in steels, have stated that the specific trapping rate for small vacancy clusters (up to 10 vacancies) is directly proportional to the number of vacancies in the cluster. Thus, the precise estimation of the defect concentration may be unequivocal only when the size of the analyzed defect is known. However, referring to *κ* can give relative information on the concentration of similar defects in the same material.

### 2.3. Transmission Electron Microscopy

Transmission electron microscopy (TEM) observations were conducted on a probe-corrected Titan STEM/TEM instrument (Thermo Fisher Scientific Inc., Waltham, MA, USA) operated at 300 kV and Titan Themis STEM/TEM (Thermo Fisher Scientific Inc., Waltham, MA, USA) instrument operated at 200 kV and equipped with a SuperX EDX System. Before introducing the sample into the microscope column, hydrocarbon contaminants were removed from the sample surface using a plasma cleaner. The Crystal Maker program was used for computer simulations of electron diffraction.

## 3. Results

**Figure A2:**
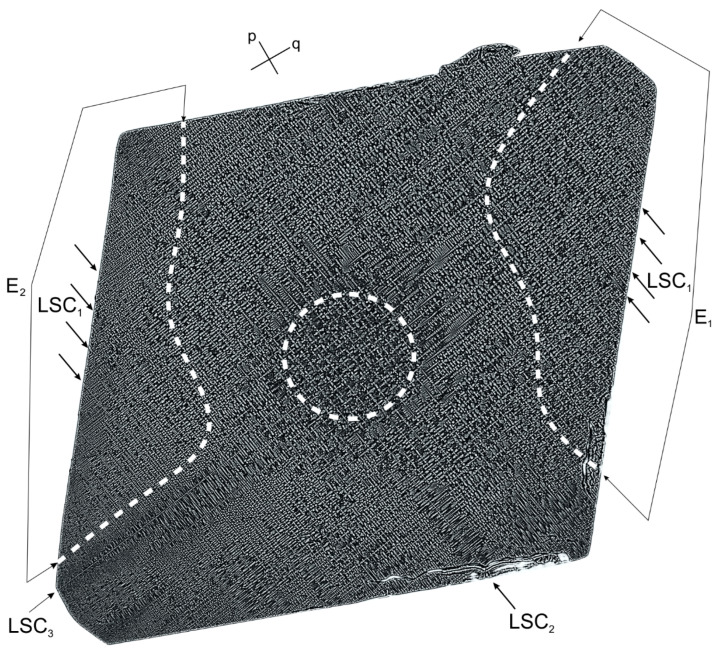
Macro-SEM image obtained from the O–P surface; p and q—the directions of dendritic arms; E_1_ and E_2_—areas of thinner secondary dendrite arms distribution; LSC_1_–LSC_3_—consecutive sets of straight chains.

Initially, the macro-SEM images of the T1 sample obtained from the O–P surface were carried out. Two systems of perpendicular secondary dendrite arms parallel to the p and q directions were found (Figure A2 in Appendix A). It was noticed, that in the sample’s middle part, which corresponds to the selector extension (SE) area (circular area, Figure A2), the dendrite arms were rather randomly distributed. However, around the SE area the dendrite arms start to arrange in straight chains (SCs). In the other part of the sample, the distribution of the secondary dendrite arms parallel to p and q appears. However, moving towards the samples edges, the dendrite arms arrange themselves in long straight chains (LSCs). Some of the longest LSCs were indicated as LSC_2_ (parallel to p) and LSC_3_ (parallel to q) in Figure A2. Moreover, two areas with outstandingly thinner dendritic structure emerge at two sides of the sample. In these areas, marked by white dotted lines and ascribed to as E_1_ and E_2_, respectively, the dendrite arms were arranged in long straight chains (LSCs) LSC_1_, which are parallel to the p direction.

### 3.1. X-Ray Diffraction Topography

The macroscopic investigation of the dendritic structure was performed basing on the obtained X-ray topograms of the ABCD (a) and A′B′C′D′ (b) sample surfaces using 113 reflections (Figure 5). Based on the topogram in Figure 5a, illustrates that the dendrites laterally grow from the SE area during the transition from the selector to the root (like in Refs. [6,9,14]). The microstructure in Figure A2, where long straight chains of secondary dendrite arms starting around the SE area can be observed, also confirms this. Laue diffraction patterns allowed us to determine crystallographic directions to which contrast bands were parallel. A pair of topograms on Figure 5a,b from the ABCD and A′B′C′D′ surface allowed for the location of orientation defects. Additionally, the topograms obtained from the surfaces ABCD and A′B′C′D′ show three blocks: I, II, and III (Figure 5c,d). In the diagrams (Figure 5c,d), the secondary arms of the dendrites are marked, which in one case have a lighter color and in another darker one. The contrasts of surfaces A′B′C′D′ and ABCD exhibit an inverse relationship: when the intensity of contrast in a particular region of the ABCD surface increases, the contrast in the corresponding area of the A′B′C′D′ surface decreases. The principles underlying this contrast inversion are discussed in Refs. [6,29].

In two cases, however, the same colors of the bands can be noticed, which may suggest the occurrence of internal stresses. These may be related to the change in the concentration of alloying elements, dendrite bending during lateral growth from the SE area or, according to Ref. [9], to the formation of vacancy clusters in areas containing low-angle boundaries—vacancy clusters may form as a result of vacancies diffusing to dislocations or through the Kirkendall effect involving alloying elements in the γ-phase, as detailed for nickel-based superalloys in Ref. [33] (similar remarks were observed in Ref. [29]). On this basis, 6 points were designated for PALS measurements (Figure 5e): 1. selector; 2. low-angle boundary; 3. low-angle boundary with secondary dendrite arms; 4. secondary dendrite arms; 5. area with internal stresses; 6. “undefected” area (with a homogeneous background).

### 3.2. Positron Annihilation Lifetime Spectroscopy

**Figure 6:**
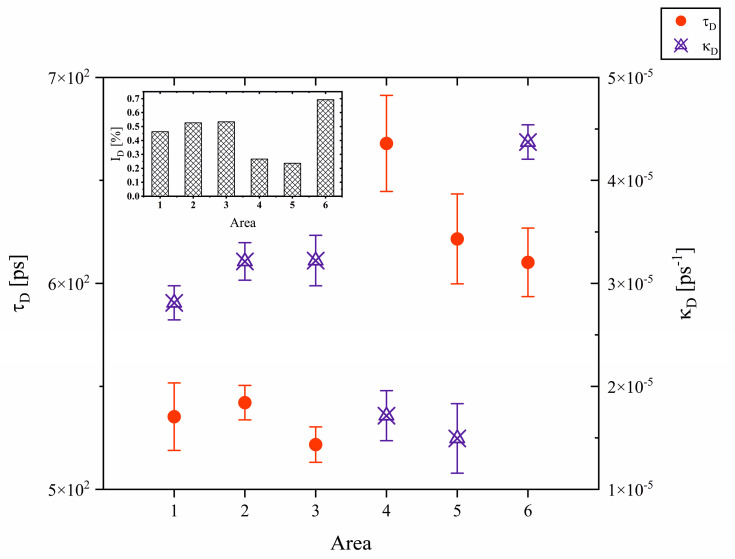
Positron lifetimes $\tau_{D}$ and annihilation rates $\kappa_{D}$ in defects determined in the selected areas of investigation 1–6. The corresponding intensities of the lifetime components $\tau_{D}$ are shown in the inset.

The positron lifetime in the bulk material of the CSMX-4 alloy under investigation was found in the range of 126 ± 2 ps. It was further assumed that the lifetime value in the bulk material is common for the whole material and set as *τ_f_* = 126 ps. This value is close to 127 ps, as reported by Dryzek et al. [34] for a CMSX-4 nickel superalloy containing W and Re. The second, longer lifetime component *τ_D_* shall be ascribed to the annihilation of positrons in three-dimensional vacancy clusters. The lifetime value of this component ranges from 522 ± 9 ps in area 3, where the low-angle grain boundary and secondary dendritic arms were identified up to 668 ± 23 ps in area 4, with the majority of secondary dendritic arms (Figure 6).

Moreover, it can be realized that the lifetime of the $\tau_{D}$ component is considerably smaller in areas 1, 2, and 3 compared to areas 4 to 6. Areas 2 and 3 are located at the low-angle grain boundary, and thus, the lifetime spectra could be affected by an impact coming from additional defects, e.g., those concerned with dislocations. Since dislocations are shallow traps for positrons, they merely localize in defects such as jogs on dislocation lines or vacancies that can be found in their vicinity. Their lifetimes will be smaller than for vacancies because of these defects’ lower symmetry than mono-vacancies [34]. Thus, it can be predicted that a fraction of positrons annihilate at dislocation-related defects, thus reducing the lifetime of the long component according to the relation [35]:

$$\lambda_{avg}=\frac{\lambda_{i}I_{i}+\lambda_{\left(i+1\right)}I_{\left(i+1\right)}}{I_{i}+I_{\left(i+1\right)}}$$

where $\lambda_{avg}=\tau_{avg}^{-1}$, $\lambda_{i}=\tau_{i}^{-1}$,$\;\lambda_{\left(i+1\right)}=\tau_{\left(i+1\right)}^{-1}\;$ and *I_i_*, *I*_(*i*+1)_ are, respectively, the annihilation rates, lifetimes and intensities for the *i*-th and the consecutive (*i* + 1) defects influencing the average annihilation rate $\lambda_{avg}$ which equals to the reciprocal of the lifetime $\tau_{avg}$. The same explanation applies to the selector area (area 1). From this area begins the undistributed dendritic growth. Thus, small structural defects could persist before they diffuse to larger complexes of free space in the material. For example, Krawczyk et al. reported in their work [9] that only defects of mono-vacancy size, with lifetimes ranging from 178 ps up to 185 ps, were found in the selector extension area in a CMSX-6 Ni-based superalloy. Therefore, the concentration of defects, demonstrated by the positron trapping rate *κ_D_* and intensity *I_D_* of the lifetime component presented in Figure 6 and its inset, respectively, are considerably higher than in areas 4 and 5 (the outstanding values registered in area 6 will be addressed later).

Further deconvolution of the defects’ lifetime, e.g., using the three-state trapping model, didn’t provide much effort. As already stated by Skoczylas et al. [36], an overestimated analysis of positron lifetime spectra with close lifetime components can provide misleading results. Components assumed to originate from several sources with short lifetime differences are, in fact, hardly traceable using conventional positron lifetime spectroscopy techniques.

The longest positron lifetime was recorded in area 4, with a high population of secondary dendritic arms observed on topography. That implies that the dendritic arms are regions preferable for the formation of vacancy clusters. As reported for Al-based alloys [37], these clusters can further agglomerate with diffusing monovacancies.

It is accepted in the literature that the positron lifetime in three-dimensional vacancy complexes increases linearly in clusters containing up to ca. 20 vacancies [38]. For larger vacancy clusters, the growth slows down to achieve a plateau at ∼0.5 ns [39,40]. It is explained by the fact that the average electron density inside the cavities decreases. Thus, the positron merely localizes at the cavity’s surface and does not longer reflect its size. However, lifetimes up to 0.595 ns for very large voids with a mean diameter of 4 nm in neutron-irradiated molybdenum were reported by Cotterill et al. [41]. As the authors have suggested, big lifetimes could be possible because the positrons trapped in voids can exist with greatly reduced electron overlap. Zaleski et al. also reported positron annihilation lifetimes ranging from 0.41 to 0.91 ns, with a rather small intensity of about 1% for several metal alloys [42]. These lifetime components were ascribed to the presence of microcracks.

The registered positron lifetimes are considerably shorter than those measured by Krawczyk et al. [10]. However, it has to be stated that the single crystal for their study was obtained with a withdrawal rate of 5 mm/min, whereas the single crystal examined in our work was produced with a 5 times slower rate, i.e., 1 mm/min. However, in the works by Gancarczyk et al. [25,26], the average size of pores in the CMSX-4 alloy grows with a lowering of the withdrawal rate, staying in evident contrast with our results. Thus, it would be valuable to perform detailed studies of positron annihilation lifetimes in these single-crystalline alloys obtained with various withdrawal rates and decide whether these registered in our work and those presented by Gancarczyk et al. [25,26] have the same origin.

We assume that, in our case, the long lifetime values are connected to the creation of microvoids at the dendrite-matrix interphase. The inter-dendritic regions are available for fluid flow during the solidification process, as shown by Madison et al. [43] in a three-dimensional model of the solidification of a Ni-based single crystal. Thus, the fluid metal is confined between the solidified dendritic connections and shrinks upon the successive liquid-to-solid transition [44]. Longer lifetimes in areas 5 and 6 could also be explained that way. Area 5 is located directly at straight chain LSC_3_, whereas area 6 covers the space of LSC_1_. In SCs, the secondary dendrite arms parallel to one direction are arranged in exceptional arrays, forming uniformly distributed inter-dendritic channels available for liquid flow. During further growth, these dendritic arms become intersected by perpendicular dendritic arms, forming confinements for the liquid metal. Thereby higher concentration of voids in area 6, demonstrated by outstanding *κ_D_* and *I_D_* parameters, can be related to the finer dendritic structure identified in area E_1_ of the sample. Thus, the size of identified micro-voids should be related to the investigated material’s thermal contraction rather than the size of inter-dendritic cavities. This hypothesis needs, however, further experimental verification.

A second explanation of the long positron lifetimes is based on the electronic structure of alloying elements. Recently, Liu and coworkers [45] explained the long lifetimes in W_x_V_1−x_O(B) nanorods (with x = 0.00 to 0.05), which were additionally growing from 488.4 ps up to 854.8 ps with increasing W content, by the higher valence state of W comparing to V (valence state was found to be W6+ and V4+). The replacement by high valence atoms leads to the production of negatively charged cation vacancies to maintain charge balance. Obviously, in this case, the probability of annihilation of positively charged positrons with an electron will be lower, and the lifetime inside the negatively charged vacancy clusters will be elongated. The presence of such alloying elements as W, Ta, Mo, and Cr, characterized by relatively high positive values of main oxidation states, could elongate the lifetime of positrons trapped in large vacancy clusters.

Whether one of these hypotheses is valid would require investigations of specimens obtained utilizing different temperatures and speeds of material withdrawal. Also, experiments using atom probe tomography would shed more light on the problem; however, it has to be mentioned that the information coming from this method covers just a small piece of material on a nanometric scale. PALS thereby probes a larger volume of material and examines the material relatively globally. Also experiments using alternative analytical techniques like impedance spectroscopy could lead to valuable findings. Recently, this method was applied to Inconel 713LC Ni-based superalloy coated with Al and Cr-Al layers to evaluate the level of the coating homogeneity [46]. The application of this method for the CMSX alloys could probe the level of inhomogeneity of the material and could be priceless for the estimation of the manufacturing conditions on the evolution of void defects.

Recently, Kundin et al. presented a study of vacancy-induced porosity in complex alloys regarding the modeling of void formation in an AlCoCrNi/Ni alloy [47]. The simulations performed in this work mirrored structural effects investigated experimentally in a CMSX-10 Ni based superalloy in [48], which the authors referred to. One of the pivotal statements of this work was that the crystal orientation is crucial for the shape evolution of voids, mimicking the change of vacancy flow directions in differently oriented dendritic structures. This statement enables the diversification of positron lifetimes among the investigated sample to be unraveled.

With respect to these theoretical studies, similar observations were made in our experimental investigations of the CMSX-4 alloy. We can see that at point 4, where lateral growth of dendrites occurs, large dendrite arms push apart as a result of competitive growth, which is visible in the topogram in the form of bands (Figure 5) and in the microstructure (Figure A2). The microstructure additionally shows identical shapes/orientations of dendrites without a clear participation of tertiary arms. The resulting voids in the interdendritic areas could have resulted in a long lifetime of positrons in the PALS method. At point 6, a similar morphology of dendrites—well-developed secondary arms without evident participation of tertiary arms—was observed. However, at point 5, a slight increase of the positron lifetime (from 617 ps to 625 ps) was observed. In this area, smaller dendrites are observed, but also a distinct LSC_3_ band from which subsequent rows of dendrite arms grow. The change in the lifetime of positrons may be related to the change in the orientation of dendrites, as well as to the various morphology of the voids [49]. The cited work [47] also described the diffusion simulation at 1525 K and the voids formed after this process. However, our samples were cut from as-cast single-crystal blades of nickel-based CMSX-4 superalloy, which are obtained at a much higher temperature of 1793 K. It experimentally confirms the Kundin et al. model [47] that void size changes depending on the dendritic growth direction.

### 3.3. Transmission Electron Microscopy

**Figure 7:**
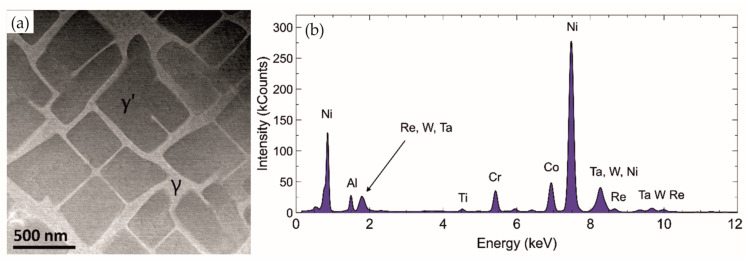
Structure of single-crystalline CMSX-4 alloy. STEM HAADF image (**a**). EDS analysis result: Ni—69.6 at. %, Co—11.5 at. %, Cr—7.2 at. %, Ta—2.0 at. %, W—2.4 at. %, Al—4.8 at. %, Re—1.1 at. %, Ti—0.8 at. %, Mo—0.6 at. % (**b**).

**Figure 8:**
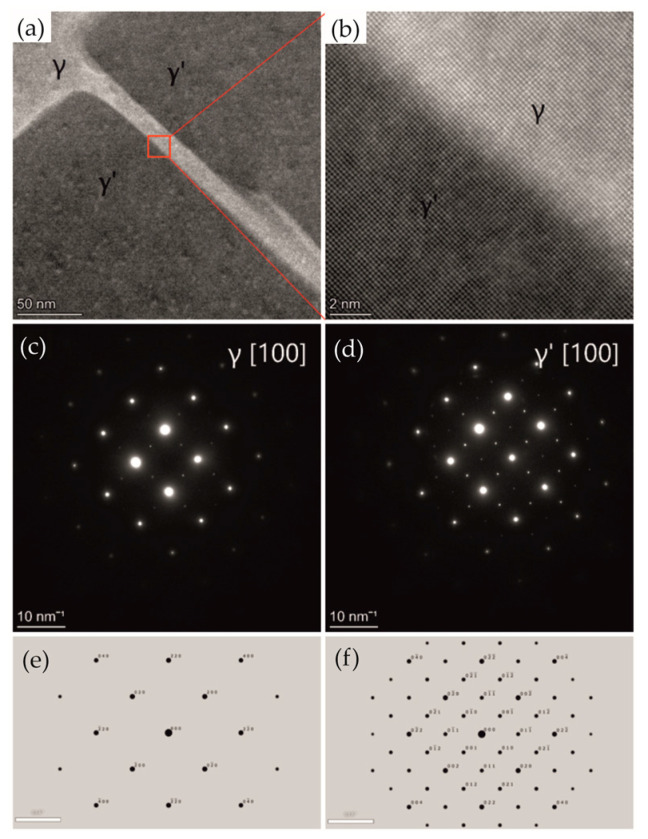
Structure of single-crystalline CMSX-4 alloy. STEM HAADF images (**a**,**b**). SAED electron diffraction pattern (**c**) with computer simulations of the γ phase in [100] zone axis (**e**). SAED electron diffraction pattern (**d**) with computer simulations of the γ′ phase in [100] zone axis (**f**).

The analysis of TEM micrographs revealed a typical γ + γ′ structure of single-crystalline CMSX alloys (Figure 7a). EDS analysis confirmed the assumed chemical composition (Figure 7b). Both phases have a similar crystal lattice and are coherent (Figure 8a,b). Electron diffraction with a selective aperture SAED (Figure 8c,d) was used for phase identification. The difference between the diffraction images concerns the presence (higher intensity) of additional reflections. The γ′ phase (Figure 8d) contains all the reflections visible in the γ phase diffraction image (Figure 8c), but additionally, there are (more clearly visible) reflections with odd and mixed indices. Figure 8e shows computer simulations of the γ phase in the [100] zone axis, while Figure 8f is a computer simulation of γ′ phase in the [100] zone axis.

**Figure 9:**
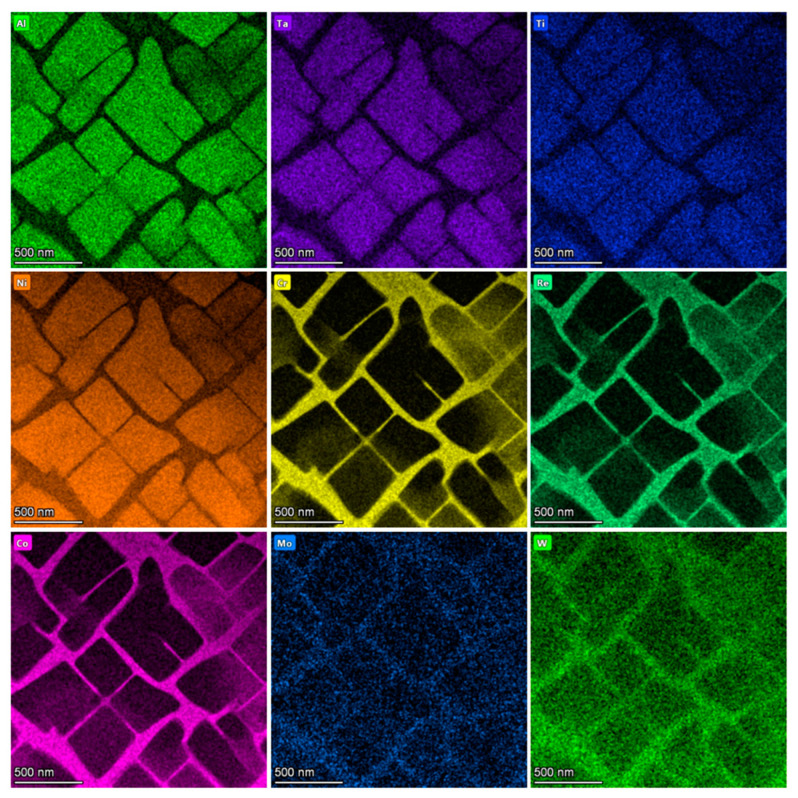
Distribution maps of Ni, Ta, Ti, Al, Cr, Re, Co, Mo and W recorded for the area shown in Figure 7a, determined using Energy Dispersive Spectroscopy EDS.

Based on the EDS investigations, it can be concluded that the distribution of elements is inhomogeneous. Increased Al, Ta, Ti, and Ni concentration occurs in γ′ grains, while in the matrix, a higher concentration of Cr, Re, Co, Mo, and W is visible (Figure 9).

**Figure 10:**
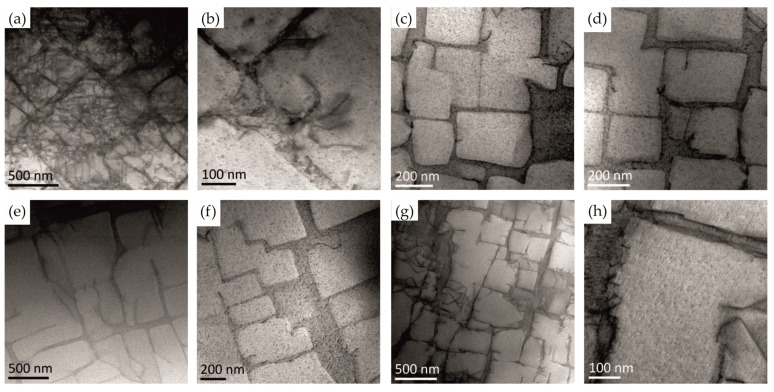
Structure of single-crystalline CMSX-4 alloy. HAADF images at area 1 (**a**,**b**), area 2 (**c**,**d**), area 3 (**e**,**f**), area 4 (**g**,**h**).

The bright field HAADF images of the structure presented in Figure 10a–h performed in different areas of the specimen revealed the presence of dislocations preferably oriented in the selector area (a, b). Nevertheless, no evidence of any micro-cracks was found; however, structural defects of 2–5 nm in size were observed in the whole specimen. Assuming the spherical shape of these defects the mean area of the plane section is ranging from ca. 12 to 80 nm^2^, about six orders of magnitude less than reported by Gancarczyk et al. [25]. However, larger pores were not detected in our material.

## 4. Conclusions

The combination of X-ray diffraction topography, positron annihilation lifetime spectroscopy, and transmission electron microscopy facilitated the identification of defects in the root of CMSX-4 single-crystal turbine blades. A comparative analysis of the results from both methods led to the following conclusions:

1. The lateral growth of dendrites starts from the selector extension area and proceeds along main paths, forming extended straight chains.
2. X-ray topography studies on thin samples allowed the observation of contrast inversions and areas where internal stresses occur.
3. The registered positron lifetimes ascribed to defects in the alloy under investigation, ranging from 522 ± 9 ps to 668 ± 23 ps, are outstandingly higher than those of typical defects found in metals and their alloys reported in the literature. These long lifetimes were ascribed to cationic vacancy clusters, which probably occur at the dendrite-matrix connection originating from the shrinking of liquid metal enclosed in inter-dendritic regions during solidification.
4. Shorter lifetimes were registered in regions where low-angle grain boundaries were identified according to microscopic results and in the area where the selector, from which the crystal growth begins, was positioned. Thus, the lifetime will be affected by short-living components related to dislocations, which are structurally connected with low-angle grain boundaries, as well as at mono-vacancies and small vacancy complexes related to primary stages of crystal growth.
5. Larger secondary dendrite arms facilitate void formation, extending the positron lifetime. Additionally, depending on the orientation of the dendrites, voids of varying sizes can be formed. The experimental results align with the theoretical model developed by Kundin et al. [47], which considers the critical role of crystallographic orientation in vacancy flow and its influence on the shape and distribution of voids in interdendritic regions. This model assumes that phenomena related to vacancy flow are strongly dependent on the dendritic structure, a conclusion that has been validated in the present study.
6. It was found that lowering the withdrawal rate from 5 mm/min to 1 mm/min results in a reduction of positron lifetimes from 1.5 ns to ca. 0.5–0.7 ns, respectively. This is probably the result of shorter void defects in the slowlier withdrawn single crystal. However, unequivocal description of this phenomenon requires additional experimentation involving alternative analytical techniques such as impedance spectroscopy.
7. TEM investigations complained with EDS analysis reviled [100] oriented γ and γ′ phases with well dispersed alloying components with low values of oxidation states. The bright field HAADF images revealed the presence of 2–5 nm structural defects in the investigated material.

## Data Availability

The original contributions presented in this study are included in the article. Further inquiries can be directed to the corresponding authors.

## Appendix A

**Table A1:** The chemical composition of the CMSX4 alloy in wt. % [1].

Ni	Co	Cr	Ta	W	Al	Re	Ti	Mo	Hf
60.7	9.6	6.5	6.5	6.4	5.6	3.0	1.0	0.6	0.1

**Table A2:** Mechanical and physical properties of CMSX-4 [50].

Property	Unit	Value
Density	Kg/m^3^	8700
Elastic modulus	GPa	250
Thermal conductivity	W/m·K	11
Melting temperature	°C	1350–1420
Coefficient of thermal expansion	μm/mK	13
0.2% proof yield strength at 760 °C	MPa	980
Ultimate tensile strength	MPa	1250
Elongation	%	10
Reduction of area	%	20
